# Biochar application alters soil properties and microbial gene profiles in a continuous cassava cropping system

**DOI:** 10.7717/peerj.21731

**Published:** 2026-09-28

**Authors:** Yanmei Zhu, Jianbing Zhang, Yundong Wei, Shiyi Zhou, Xingming Qin

**Affiliations:** 1 Key Laboratory of Environment Change and Resources Use in Beibu Gulf, Ministry of Education, Nanning Normal University, Nanning, China; 2 Guangxi Geographical Indication Crops Research Center of Big Data Mining and Experimental Engineering Technology, Nanning Normal University, Nanning, China; 3 Guangxi Key Laboratory of Earth Surface Processes and Intelligent Simulation, Nanning Normal University, Nanning, China; 4 Guangxi Subtropical Crops Research Institute, Nanning, China

**Keywords:** Cassava continuous monoculture, Rhizosphere microbiome, Metagenomic sequencing, Microbial functional potential, Soil carbon and nitrogen cycling

## Abstract

Continuous cassava cropping can lead to soil degradation and disturbances in soil microbial functions, threatening the sustainability of cassava production systems. To address these challenges, this study examined the effects of biochar on soil physicochemical properties and microbial functional profiles in a continuous cassava cropping system. Two biochar application rates (0 and 3 Mg ha^−1^) were applied, and metagenomic sequencing was conducted to evaluate microbial community composition and functional genes related to carbon and nitrogen cycling in rhizosphere and bulk soils. The results demonstrated that biochar was associated with higher soil pH, soil organic matter, and available nutrient content, with a stronger effect in rhizosphere soil. Biochar application altered the genetic potential of microbial communities, particularly in the rhizosphere soil. In terms of functional categories, biochar was related to higher gene abundances in “homologous recombination” and “DNA replication” categories (Kyoto Encyclopedia of Genes and Genomes (KEGG) database), as well as the “replication, recombination, and repair” category (evolutionary genealogy of genes: Non-supervised Orthologous Groups (eggNOG) database) in rhizosphere soil. Biochar also affected the abundance of carbon cycling genes, particularly in the rhizosphere. The abundance of the aerobic respiration-related gene *coxA* was increased, while the abundance of the anaerobic fermentation gene L-lactate dehydrogenase (*LDH*) was decreased. Additionally, in rhizosphere soil, biochar significantly increased the abundance of *norB* (denitrification), *GDH2* (nitrogen mineralization), and *nifD* (nitrogen fixation), while decreasing the abundance of genes involved in nitrogen assimilation (*gltB*), assimilatory nitrate reduction (*nirA*), nitrogen mineralization (*cynS*), and nitrogen uptake (*nrtA, nasF, cynA, nrtC, and nasD*). Together, these results suggest that biochar application may enhance nutrient availability and reshape microbial functional potential primarily in the cassava rhizosphere, providing field evidence for biochar use in continuous cassava cropping.

## Introduction

With the continuous development of agricultural production, particularly in high-intensity farming systems, soil quality degradation has become a serious global problem (Li et al., 2023). In continuous cropping systems, soil acidification, nutrient imbalances, and the reduction of organic carbon have become major bottlenecks restricting sustainable agricultural production (Han et al., 2024; Reeves, 1997; Wang et al., 2024b; Zhang et al., 2016). Traditional farming practices and monoculture cropping systems have led to the deterioration of its physical and chemical properties and an imbalance in microbial community structure (Li et al., 2022a; Liu et al., 2024b). These changes ultimately affect soil health, plant growth, and agricultural productivity (Kaushal et al., 2022). To address these challenges, biochar, as a soil amendment material, has attracted widespread attention in the agricultural sector. Biochar is a carbon-rich substance produced by pyrolyzing agricultural waste, forestry residues, or other organic materials under low-oxygen conditions (Di Domenico et al., 2024). Due to its porous structure and stable chemical properties, biochar not only improves soil pH and physical structure but also helps maintain organic carbon levels in the soil over the long term, reducing organic carbon loss (Sun et al., 2022). The high specific surface area of biochar allows it to adsorb and store significant amounts of nutrients, thereby enhancing the soil’s nutrient retention capacity and fertilizer use efficiency (Ramesh & Raghavan, 2024; Zhang et al., 2023b). Additionally, biochar can regulate soil microbial activity, increase microbial diversity and functionality, and promote the recovery of soil health (Xu et al., 2023; Zhang & Shen, 2022). However, although biochar has many beneficial effects on soil improvement, its application may also cause some adverse impacts. Previous studies have shown that excessive biochar addition can disrupt soil nutrient balance and increase soil salinity, which may negatively influence crop growth (Vijay et al., 2021; Tang et al., 2022). Therefore, the application of biochar in agricultural systems should be carefully evaluated and properly managed.

Soil carbon and nitrogen cycles involve the transformation of soil carbon and nitrogen between different forms (Zhou et al., 2024). These processes play a crucial role in soil health and are directly linked to the productivity and stability of agroecosystems (Elbasiouny & Elbehiry, 2019; Van Rijssel et al., 2025). Soil microorganisms are the primary drivers of soil carbon and nitrogen cycling (Kuypers, Marchant & Kartal, 2018). Under fertilization management, certain microbial groups, notably *Actinobacteria* and *Acidobacteriota*, are particularly important for soil carbon and nitrogen transformations (Liu et al., 2024b). Manure fertilization can shift the relative abundance of these communities and alter the expression of nitrogen-related functional genes, such as *nifH, napA* and *amoA*, compared with soils that receive no fertilizer (Li et al., 2022b). These changes highlight how fertilization affects both the composition and functional potential of soil microbial communities. Such changes in microbial communities may also affect soil carbon and nitrogen cycling, potentially altering the balance of these processes. Imbalances in soil carbon and nitrogen cycling may further accelerate nutrient losses and increase greenhouse gas emissions, thereby negatively affecting soil ecosystem functioning and agricultural sustainability (Ghaley, Porter & Sandhu, 2014). In continuous cropping systems, disruptions in microbial-mediated carbon and nitrogen transformations can also reduce nutrient use efficiency and impair long-term soil health (Ding et al., 2024). Therefore, understanding the microbial mechanisms involved in soil carbon and nitrogen cycling is important for developing sustainable soil management strategies.

Cassava (*Manihot esculenta* Crantz) is an important economic crop in tropical and subtropical regions, particularly in Africa and Asia, where it is widely cultivated in nutrient-poor soils (Sinta & Dansa, 2023). However, the continuous cultivation of cassava leads to sustained soil degradation, particularly with respect to the limited availability of key nutrients, which severely impacts cassava growth and yield (Chen et al., 2025; Sinta & Dansa, 2023). Therefore, improving soil health in cassava fields, restoring soil fertility, and promoting sustainable production have become key issues in current agricultural research. Many studies have shown that biochar can substantially improve soil health and boost crop yields. These findings are mainly based on field observations from various cropping systems, including temperate farmland such as maize fields, semi-arid alkaline soils, and rice paddies (An et al., 2022; Wang et al., 2024c; Zhang et al., 2020). Although biochar has been shown to improve soil properties, studies addressing its effects on the abundance of carbon- and nitrogen-cycling genes in cassava fields, based on metagenomic analysis, are still scarce. In the same experimental system, 16S rRNA gene sequencing previously revealed biochar-induced changes in bacterial community composition in cassava soils (Zhu et al., 2026). These findings provided insights into microbial responses at the taxonomic level, but whether these community shifts were accompanied by changes in functional potential remains unclear. Therefore, metagenomic sequencing was applied in the present study to further characterize microbial functional profiles and carbon- and nitrogen-cycling genes.

The rhizosphere is the area around plant roots, where a variety of microbes thrive and influence plant growth and development (Solomon, Janda & Molnár, 2024). The rhizosphere is directly influenced by root exudates, respiration, and nutrient absorption, leading to higher nutrient turnover rates (Li et al., 2025; Wang et al., 2024a). It also exhibits more active microbial interactions and more complex plant-microbe interactions compared to non-rhizosphere soil, which is relatively stable (Handakumbura, Ubach & Battu, 2021; Mishra, Zhang & Yang, 2024). Due to these marked differences, biochar may exert distinct effects on rhizosphere and non-rhizosphere soils. Studying its differential regulatory mechanisms in these two microenvironments will help us better understand how biochar influences soil environments, microbial communities, and crop growth.

Therefore, this study aims to investigate the effects of biochar application on the physicochemical properties of rhizosphere and non-rhizosphere soils in cassava fields. Since changes in soil physicochemical properties may further affect microbial activity and nutrient transformation processes, metagenomic sequencing was used to explore the impact of biochar on soil nutrients, the functional categories of microbial community, and carbon and nitrogen cycling genes. We hypothesized that biochar-induced changes in soil properties would be associated with shifts in microbial communities and functional genes in both rhizosphere and non-rhizosphere soils. This study may improve our understanding of the microbial mechanisms underlying biochar-mediated regulation of soil carbon and nitrogen cycling in continuous cassava cropping systems.

## Materials and Methods

### Experimental site description

The experiment was conducted at the cassava research field of the Guangxi Subtropical Crops Research Institute, located at 108°20′03.41″E and 22°54′04.59″N. The site is situated in a humid subtropical monsoon climate zone, characterized by abundant sunlight, high annual rainfall, and a nearly frost-free environment with no occurrences of snow. The region has a long-term mean temperature of approximately 21.6 °C, an average annual precipitation of 1,304.2 mm, and a mean relative humidity of about 79%. The field has been continuously cultivated with cassava for over a decade, with Haplic Acrisols being the predominant soil type (Spaargaren & Deckers, 1998).

### Experimental materials

The cassava cultivar Nanzhi 199 is widely planted in southern China due to its exceptional yield, starch content, and resistance to environmental stresses (Zhang, 2025). The biochar applied in the trial was sourced from Henan Lizhe Environmental Protection Technology Co., Ltd. It was produced from maize straw through pyrolysis at 500 °C for 2 h under oxygen-limited conditions using a continuous carbonization system. The key physicochemical characteristics of the biochar are presented in Table S1.

### Experimental design and samples collection

A single-factor randomized block design was adopted for this study. Two levels of biochar application were tested, 0 and 3 Mg ha^−1^, each with three replicates. For clarity, treatments were coded as follows: CB, bulk soil without biochar application; CR, rhizosphere soil without biochar application; BB, bulk soil with biochar application; and BR, rhizosphere soil with biochar application. Cassava was planted on 24 March 2023 at a spacing of 0.9 m × 1.0 m, and each experimental plot occupied 21.6 m^2^. Biochar was incorporated into the topsoil (0–30 cm) by manual tillage prior to planting. Approximately 90 days after planting, a compound fertilizer (N: P_2_O_5_: K_2_O = 17:17:17) was applied at a rate equivalent to 412 kg hm^−2^.

Soil sampling for both rhizosphere and non-rhizosphere zones was carried out during the tuber bulking stage on 19 September 2023. For rhizosphere samples, three cassava plants were randomly selected from each plot. After gently removing loose surface soil, the whole root system was excavated including tubers. Following the removal of large soil aggregates, tubers were transferred to sterile sampling bags, and soil adhering to the root surfaces was collected using a sterilized sieve. Bulk soil was sampled roughly 0.5 m away from the selected plants using an S-shaped sampling strategy, with five subsampling points per plot. All samples were taken from the 0–20 cm soil layer. The collected soil was homogenized and then divided into two subsamples using the quartering method. One subsample was placed in an insulated container with ice packs and promptly stored at −80 °C for the extraction of DNA. The remaining soil was air-dried at room temperature for later measurements of physicochemical properties.

### Determination of soil physicochemical properties

The soil physicochemical properties of all samples were analyzed following the procedures outlined by Bao (2000). After the soil samples were air-dried at ambient temperature, visible plant residues, stones, and other impurities were removed. The samples were subsequently ground and sieved to meet analytical requirements. Soil pH was determined using a pH meter in distilled water with a soil-to-water ratio of 1:2.5. Soil organic matter (SOM) was quantified using the external heating potassium dichromate oxidation method. Alkali-hydrolyzable nitrogen (AN) was determined *via* the alkaline hydrolysis diffusion method. Available phosphorus (AP) was extracted using NaHCO_3_ and measured by the molybdenum-antimony colorimetric method. Available potassium (AK) was analyzed using the ammonium acetate extraction-flame photometry method. Total nitrogen (TN) was assessed through the Kjeldahl digestion method. Total phosphorus (TP) was analyzed using the molybdenum-antimony colorimetric technique. Total potassium (TK) content was determined using the flame photometry method. Each parameter was measured in triplicate.

### DNA extraction and metagenomic sequencing

Microbial DNA was isolated from twelve soil samples using the E.Z.N.A.^®^ Stool DNA Kit (Omega Bio-tek, Norcross, GA, USA) following the manufacturer’s instructions. Metagenomic shotgun sequencing libraries were constructed and sequenced by Shanghai Biozeron Biotechnology Co., Ltd, Shanghai, China. Briefly, one µg of genomic DNA from each sample was fragmented with a Covaris S220 ultrasonicator (Woburn, MA, USA), and sequencing libraries with an average insert size of approximately 450 bp were prepared. All libraries were sequenced on the Illumina NovaSeq 6000 platform in paired-end 150 bp mode. Raw reads were subjected to quality filtering using Trimmomatic v0.36 (https://github.com/usadellab/Trimmomatic/releases/tag/v0.36) to remove adapter sequences and low-quality bases. High-quality reads were mapped to the reference genome(s) using BWA mem algorithm (http://bio-bwa.sourceforge.net/bwa.shtml), and reads aligning to host/plant-derived sequences were removed. The remaining reads were retained as clean reads for downstream analyses.

Taxonomic assignments of clean reads were performed using Kraken2 with a customized database containing all bacterial, archaeal, fungal, viral, protozoan, and algal genomes available in NCBI RefSeq (release 20221209: https://www.ncbi.nlm.nih.gov/refseq/). Reads were classified into seven taxonomic ranks (domain to species) or labeled as unclassified. Abundance estimates at the genus and species levels were refined using Bracken to improve quantitative accuracy across closely related taxa. Relative abundance at each taxonomic level was calculated as the sum of abundances of all member species.

*De novo* assembly of clean reads from each sample was carried out using MEGAHIT (v1.1.1-2-g02102e1). Open reading frames (ORFs) were predicted from assembled contigs with METAProdigal (v2.6.3). All predicted ORFs were clustered into a nonredundant gene catalog using CD-HIT, with the longest sequence in each cluster retained as the representative gene. High-quality reads were mapped back to this gene set with BWA-MEM (v0.7.17), and gene abundances (alignment length ≥ 50 bp, identity > 95%) were normalized to transcripts per million (TPM).

Functional annotation of the nonredundant gene set was carried out using several established reference databases. Kyoto Encyclopedia of Genes and Genomes (KEGG) database assignments were obtained with Kofam v1.2.0. Functional classification at broader levels was performed using the evolutionary genealogy of genes: Non-supervised Orthologous Groups (eggNOG) v5.0 database. Annotation of carbohydrate-active enzymes was achieved by comparing the gene set with the CAZyme v8 database. To further complement these annotations, protein sequences were aligned to the NCBI NR database using DIAMOND (v0.9.22.123) based on Protein Basic Local Alignment Search Tool (BLASTP) similarity searches.

### Statistical analysis

Differences in soil chemical properties among different groups were evaluated using one-way analysis of variance (ANOVA) performed in SPSS version 26.0 (SPSS Inc., Chicago, IL, USA). When significant effects were detected, Duncan’s multiple range test was applied for *post hoc* comparisons. Statistical significance was defined as *P* < 0.05. All values are presented as the mean ± standard error. Pearson correlation analysis was used to examine the associations among environmental variables and the abundances of functional genes. Data visualizations were generated through an online analysis tool (https://cnsknowall.com/#/Home/Contain/BottomContainAll).

## Results

### Effects of biochar application on the soil physicochemical properties in the cassava field

Figure 1 represents the soil physicochemical properties under the different groups. Biochar addition (bulk soil (BB) and rhizosphere soil (BR) groups) showed increasing trends in soil pH, SOM, and the available nutrient indicators (AN, AP, and AK), while the increases in AP and AK were statistically significant. These results suggest that biochar may improve soil acidity conditions and increase nutrient availability in the cassava field. In this study, SOM and AN were highest in BB, while AP and AK reached their highest levels in BR. By comparison, rhizosphere soil without biochar application (CR) showed lower values for most of the available nutrient indicators. No significant differences were detected in TN, TP, or TK across the groups. Overall, the findings show that biochar appears to be linked to improved soil nutrient status in rhizosphere and bulk soils, with contrasting responses observed between the two compartments.

**Figure 1:**
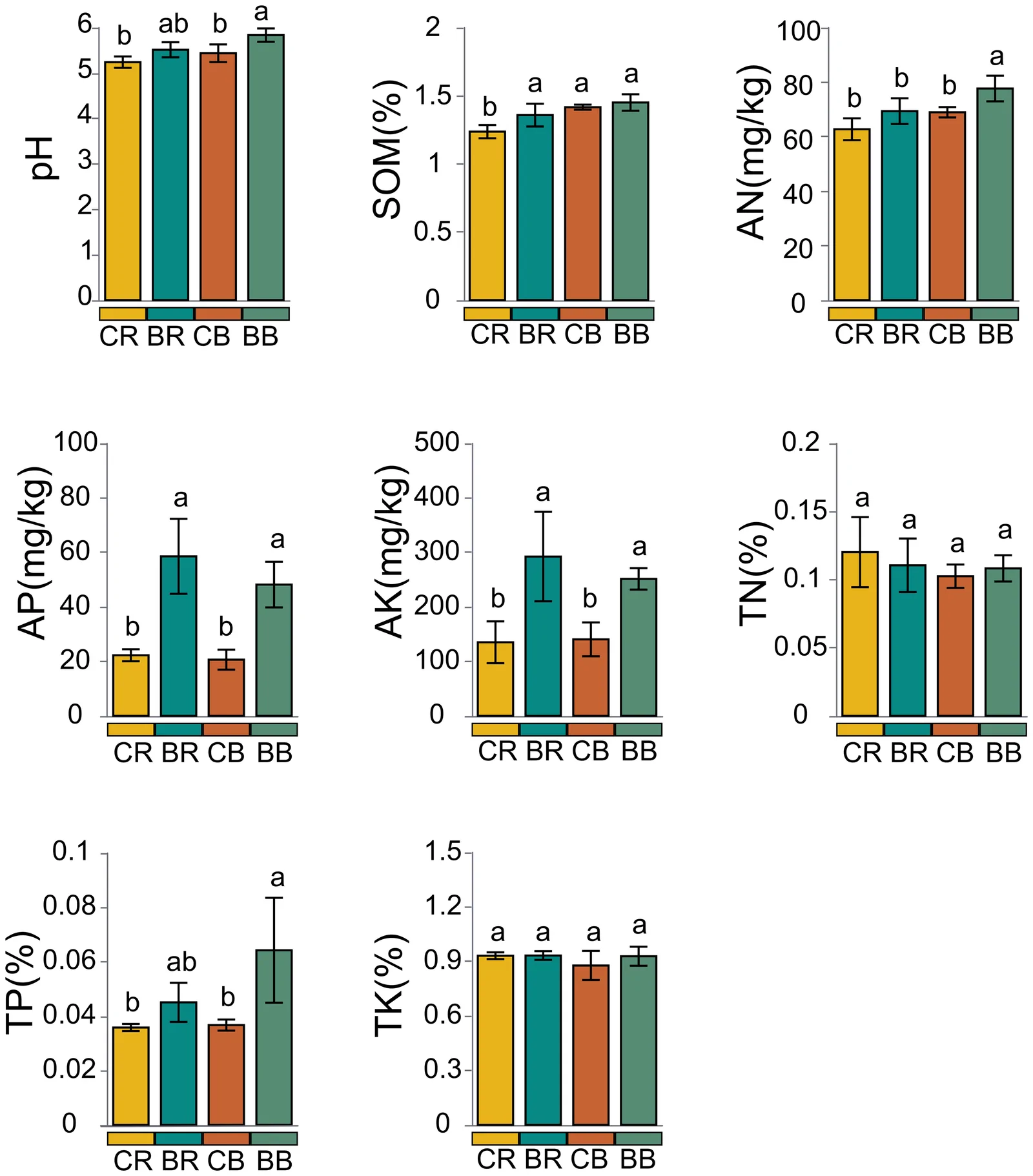
The physicochemical properties under different groups. CR, rhizosphere soil without biochar application; BR, rhizosphere soil with biochar application; CB, bulk soil without biochar application; BB, bulk soil with biochar application; pH, hydrogen ion concentration; SOM, soil organic matter; AN, alkali hydrolyzable nitrogen; AP, available phosphorus; AK, available potassium; TN, total nitrogen content; TP, total phosphorus; TK, total potassium. Different lowercase letters represent significant differences (*P* < 0.05).

### Metagenomic sequencing information and microbial community composition

Twelve libraries were sequenced using the Illumina NovaSeq platform (Illumina, San Diego, CA, USA). On average, 74,335,860 reads of raw sequencing data were generated, and 69,461,045 reads of high-quality clean data were retained after quality control (Table S2). The application of biochar had different effects on the gene abundances of bacteria, eukaryotes, and archaea of the soil. Among the bacterial communities, bulk soil without biochar application (CB) showed the highest gene abundance, while BR had relatively lower abundance. Biochar application slightly reduced the gene abundances of eukaryotes and archaea, but no significant differences were observed between the groups (Fig. 2A). Although biochar application tended to increase soil microbial species diversity, the difference was not significant. Non-metric MultiDimensional Scaling (NMDS) analysis further revealed differences in the microbial community structure between treatments. As shown in Fig. 2C, NMDS ordination showed a separation tendency among groups in two-dimensional space, suggesting that biochar application may be associated with changes in the microbial community structure of cassava field soils.

**Figure 2:**
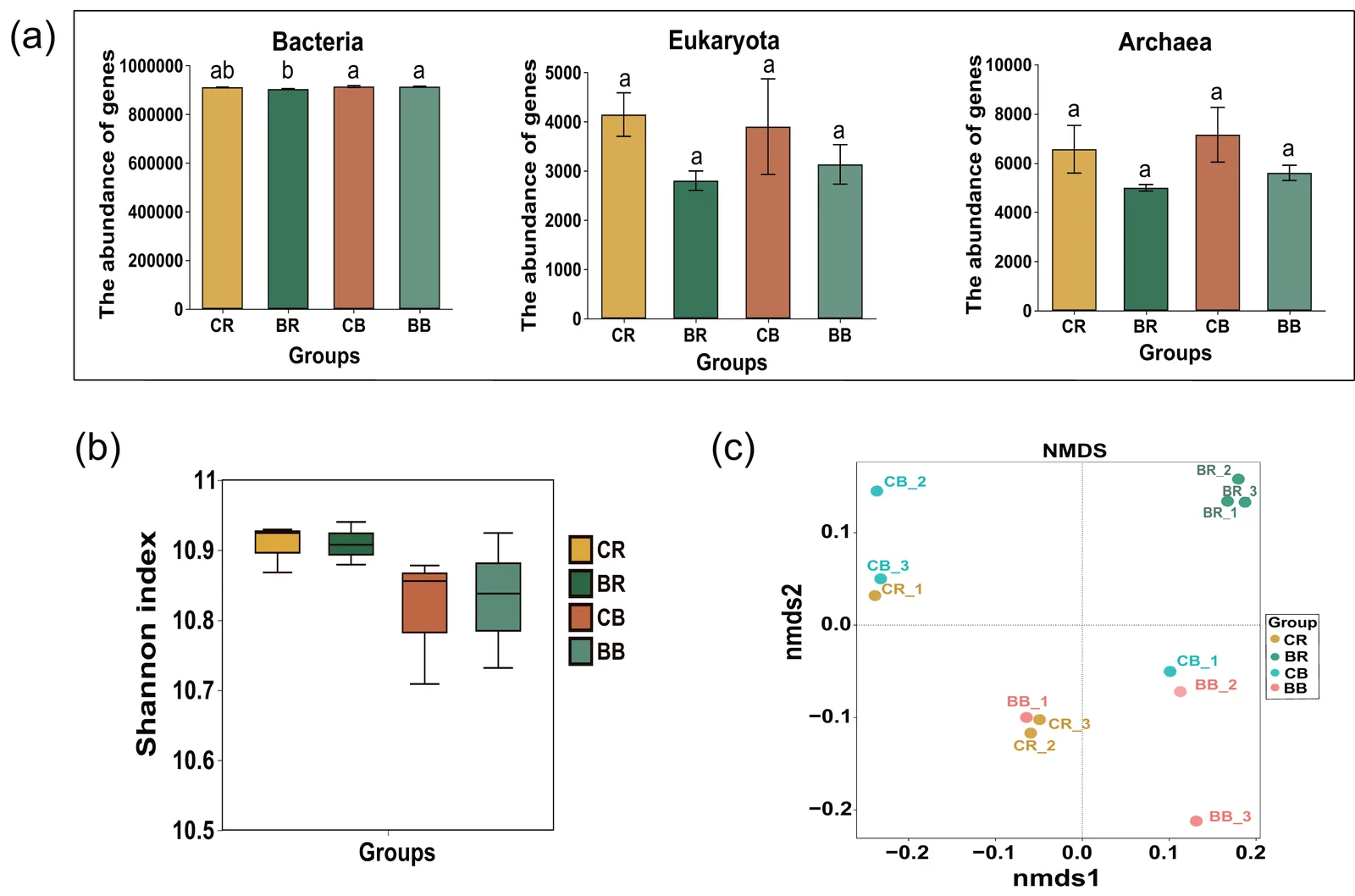
The soil microbial community composition and diversity. The gene abundance of bacterial, eukaryotic and archaeal communities across different groups (A). The comparison of shannon index among different groups (B). Non-metric multidimensional scaling (NMDS) plot for different groups (C). CR, rhizosphere soil without biochar application; BR, rhizosphere soil with biochar application; CB, bulk soil without biochar application; BB, bulk soil with biochar application. Different lowercase letters represent significant differences in the bar plot (*P* < 0.05).

### The impact of biochar application on soil microbial function in the cassava field

Gene function annotation and classification were carried out using the KEGG, eggNOG, and Carbohydrate-Active enZYmes (CAZyme) databases. The primary KEGG annotation showed that “metabolism” was the most enriched functional category (Fig. 3A). The effects of biochar on the microbiomes in the rhizosphere and non-rhizosphere soils differed. In the non-rhizosphere soil, biochar application caused significant changes in only a few KEGG functional categories, whereas more extensive alterations were observed in the rhizosphere soil (Fig. 3B). Compared to CR, BR exhibited higher gene abundance in categories such as “homologous recombination” and “DNA replication” (Fig. 3C).

**Figure 3:**
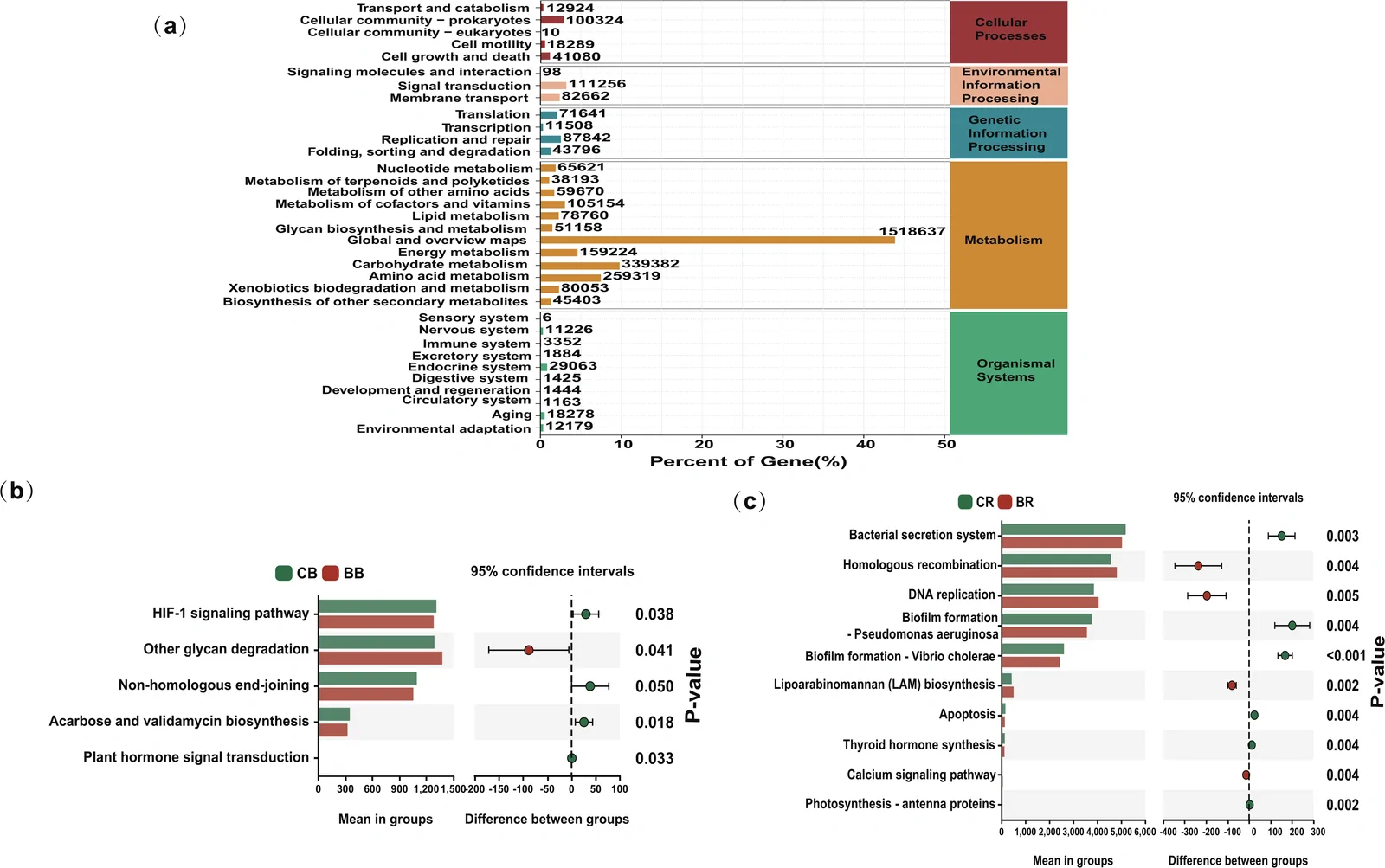
The annotation and classification of soil microbial functions in the KEGG database. The percentage of genes were annotated in the KEGG database for the primary functional annotation (A). Significantly different KEGG level 3 functional categories between the CB and BB groups (*P* < 0.05) (B). Top 10 KEGG level 3 functional categories ranked by *P* value between CR and BR (*P* < 0.05) (C). CR, rhizosphere soil without biochar application; BR, rhizosphere soil with biochar application; CB, bulk soil without biochar application; BB, bulk soil with biochar application.

Figure 4A shows the top 10 functional categories with the highest gene abundance in the eggNOG database, along with the effects of biochar on these functional categories in different microhabitats (Fig. 4B, Figs. S3, and S4). In the non-rhizosphere soil, biochar application had no significant impact on the microbial functional categories (Fig. S3). However, in the rhizosphere soil, biochar significantly reduced the gene abundance in categories such as “function unknown”, “cell wall/membrane/envelope biogenesis”, and “carbohydrate transport and metabolism”, while significantly increasing the gene abundance in the “replication, recombination and repair” category (Fig. 4B).

**Figure 4:**
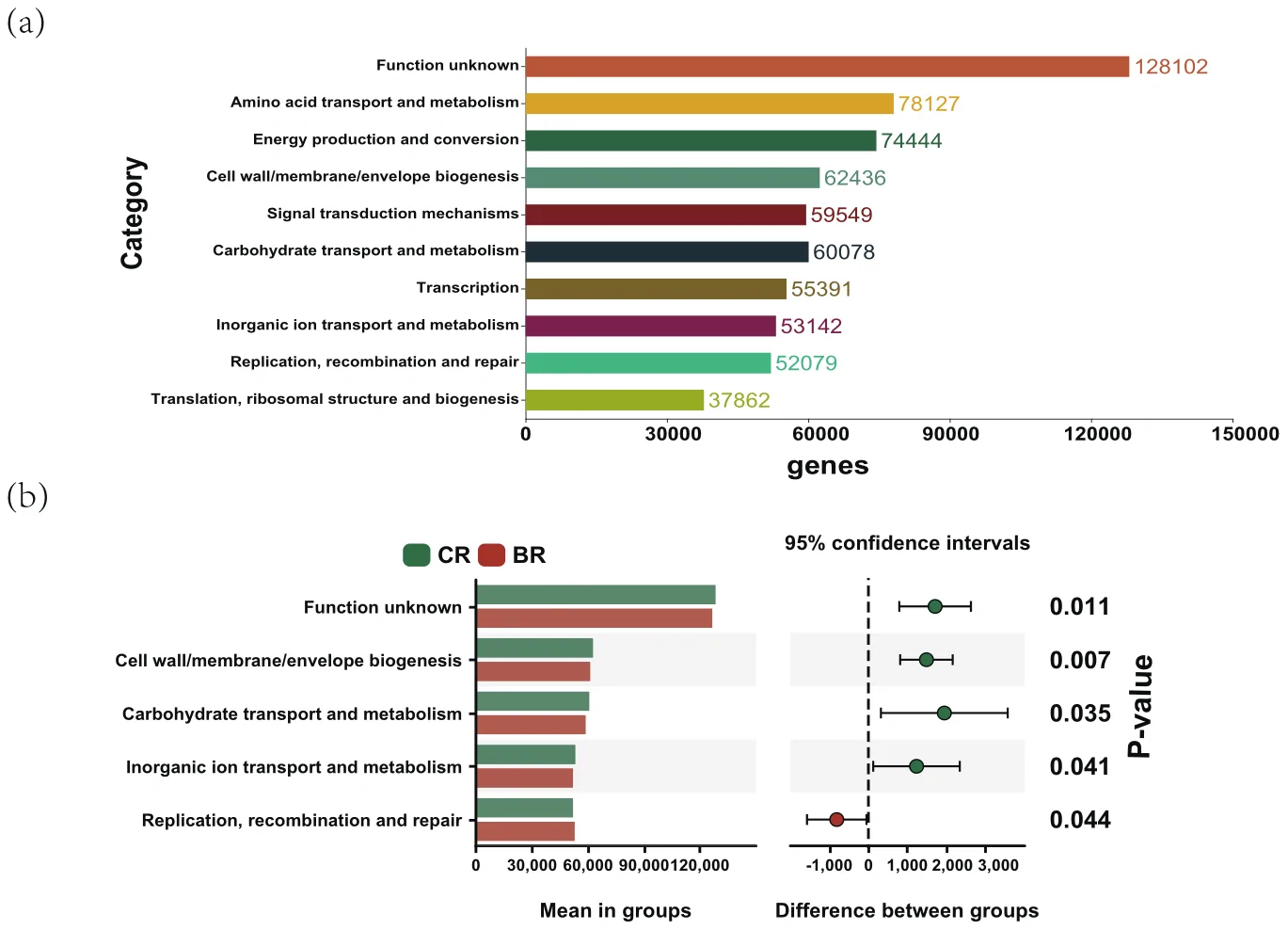
The annotation and classification of soil microbial functions in the eggNOG database. The top 10 functional categories with the highest gene abundance in the eggNOG database (A). Differentially abundant eggNOG functional categories between the CR and BR (*P* < 0.05) (B). CR, rhizosphere soil without biochar application; BR, rhizosphere soil with biochar application.

Figure 5A shows the functional annotation categories from the CAZyme database, with “glycoside hydrolases” being the most enriched category. Figures 5B and 5C display the gene abundance comparisons of the top ten functional groups with significant differences between CR and BR, and CB and BB, respectively. All of these groups showed significant differences (*P* < 0.05), with especially notable differences in the rhizosphere soil. Biochar significantly altered the functional structure of the rhizosphere soil microbial community by altering CAZyme-associated gene profiles, particularly “glycoside hydrolases”, “glycosyl transferases”, and “carbohydrate-binding modules” categories.

**Figure 5:**
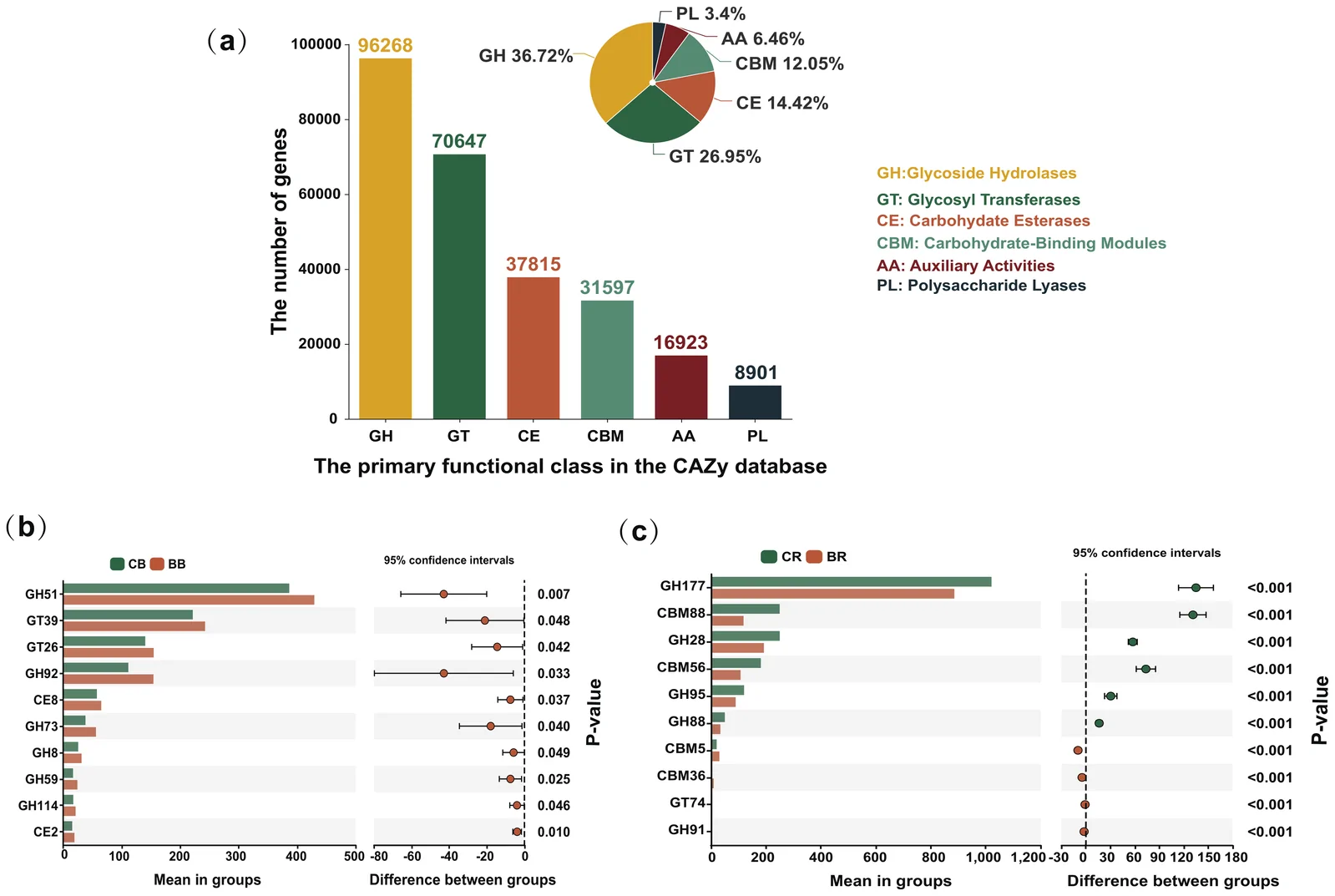
The annotation and classification of soil microbial functions in the CAZyme database. The numbers of genes were annotated in the CAZyme database (A); enzyme categories in the CAZyme database with the top 10 highest significance levels in the comparison between CB and BB (B); enzyme categories in the CAZyme database with the top 10 highest significance levels in the comparison between CR and BR (C). CR, rhizosphere soil without biochar application; BR, rhizosphere soil with biochar application; CB, bulk soil without biochar application; BB, bulk soil with biochar application.

### The effect of biochar on microbial genes involved in carbon cycling in the cassava field

A diagram of the carbon cycle was created based on KEGG database annotations (Fig. 6A), and a comparison of genes abundance related to soil carbon cycling was conducted between groups from different microhabitats (Fig. 6B). The carbon cycling functional genes examined in this study include: anaerobic carbon fixation (five genes), aerobic carbon fixation (two genes), aerobic respiration (four genes), and fermentation (one gene). No significant differences were observed in the abundance of key carbon cycling genes between BB and CB (Fig. S1), implying that biochar exerts little effect on carbon cycling gene abundance in non-rhizosphere soil. However, in rhizosphere soil, biochar application significantly increased the abundance of the aerobic respiration–related gene *coxA* and significantly decreased the abundance of the fermentation-related L-lactate dehydrogenase (*LDH*) gene.

**Figure 6:**
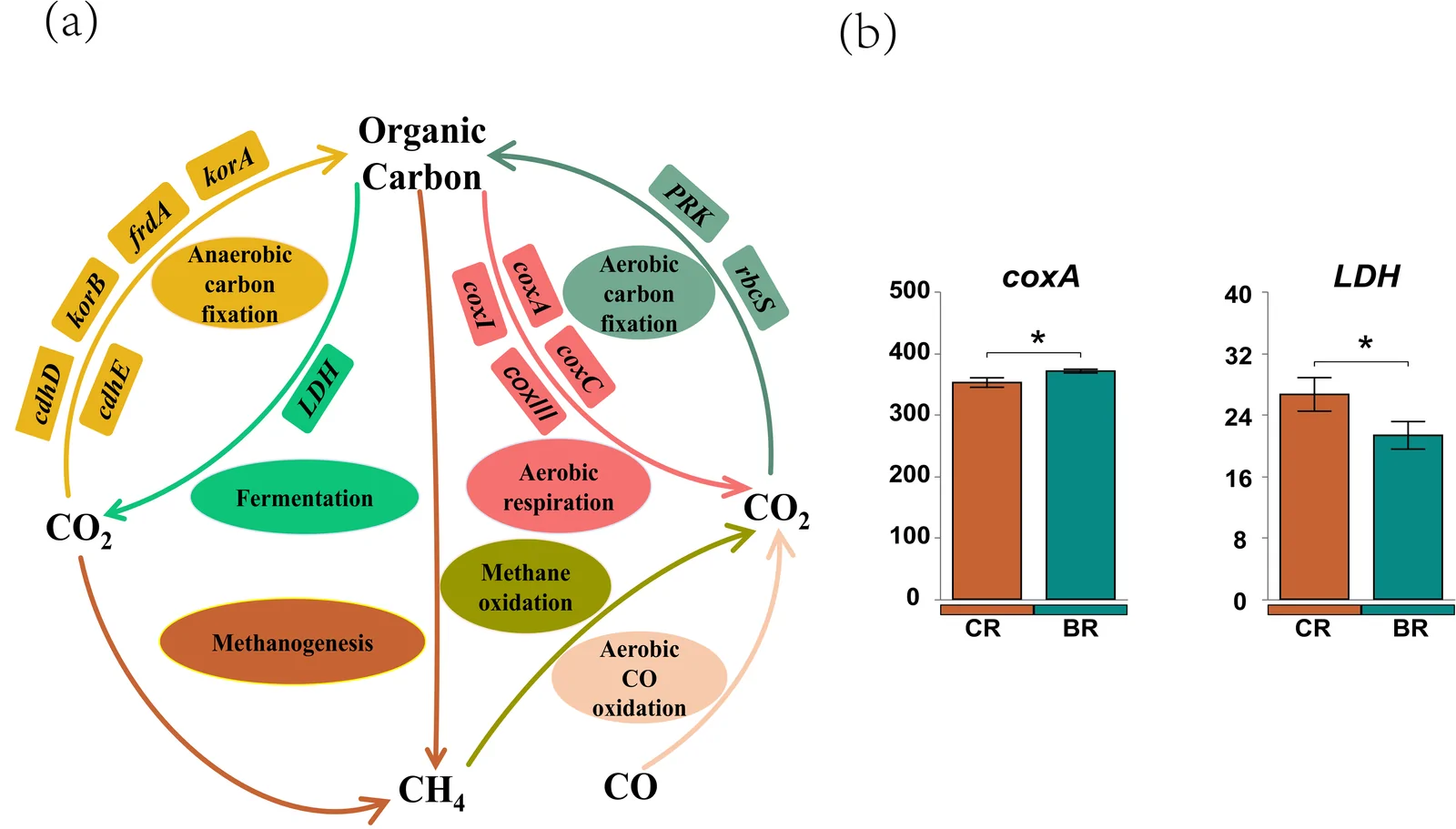
Functional genes involved in soil carbon cycling under different biochar treatments. The functional genes related to soil carbon cycling (A). The Relative abundance of significantly different carbon-cycling genes in the comparison between CR and BR (B). The contents inside the oval represent the pathways of the carbon cycle; genes are indicated in italicized font within rectangular boxes; arrows denote the direction of the pathways; the names of the pathways, related genes, and arrows are all shown in the same color. CO_2_: carbon dioxide; CO, carbon monoxide; CH_4_, methane; CR, rhizosphere soil without biochar application; BR, rhizosphere soil with biochar application. * Represents significant differences at *P* < 0.05.

### The effect of biochar on microbial genes involved in nitrogen cycling in the cassava field

This study examined the microbial genes involved in the nitrogen cycle and compared their abundance between groups from different microhabitats (Figs. S5, Fig. 7). The functional genes related to the nitrogen cycle were annotated and classified into 8 categories: nitrification (eight genes), denitrification (five genes), nitrogen fixation (three genes), assimilatory nitrate reduction (three genes), dissimilatory nitrate reduction (nine genes), nitrogen mineralization (10 genes), nitrogen assimilation (five genes), and nitrogen uptake (14 genes). The application of biochar significantly increased the abundance of the *norB* gene involved in denitrification pathway, the *GDH2* gene participated in the nitrogen mineralization pathway, and the *nifD* gene in the nitrogen fixation pathway in rhizosphere soil, suggesting that biochar promoted these nitrogen cycling processes, which contributed to microbial nitrogen transformation and fixation. Biochar application significantly decreased the abundance of the nitrogen assimilation (*gltB*) gene involved in the nitrogen assimilation pathway, the assimilatory nitrate reduction (*nirA*) gene in the assimilatory nitrate reduction pathway, the nitrogen mineralization (*cynS*) gene in the nitrogen mineralization pathway, and the nitrogen uptake (*nrtA*, *nasF*, *cynA*, *nrtC*, and *nasD*) genes in the nitrogen uptake pathway, indicating that biochar may reduce the reliance on these pathways by improving the nitrogen supply in the soil. There were no significant differences in the abundance of cycling genes between BB and CB (Fig. S6), indicating that biochar has a minimal impact on gene abundance involved in nitrogen cycling in non-rhizosphere soils.

**Figure 7:**
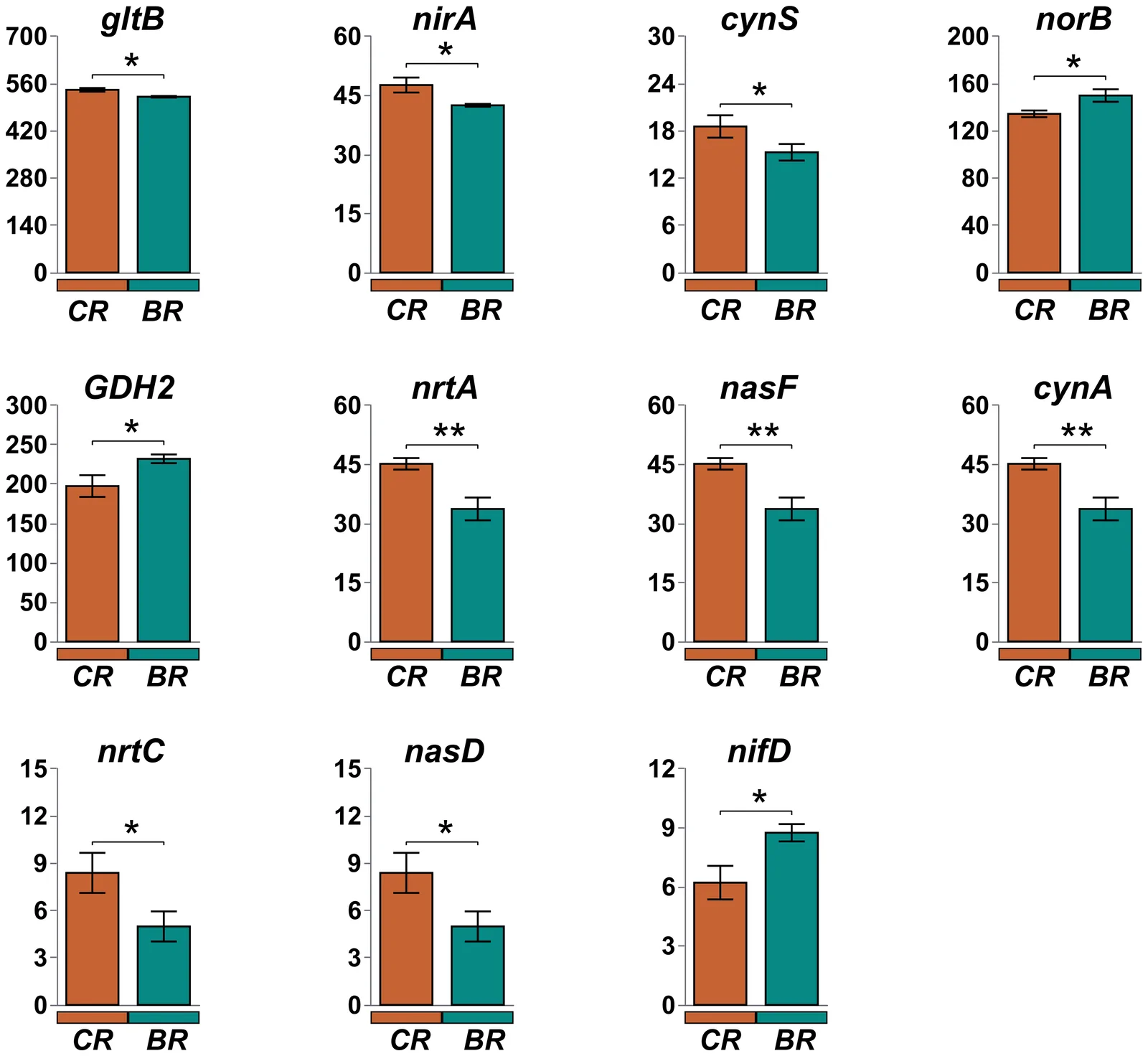
The relative abundance of significantly different nitrogen-cycling genes in the comparison between CR and BR. CR, rhizosphere soil without biochar application; BR, rhizosphere soil with biochar application. *Represents significant differences at *P* < 0.05; ** represents significant differences at *P* < 0.01.

### Correlation analysis between soil physicochemical properties and carbon and nitrogen cycling genes

Figures S7A, S7B shows the results of a Pearson correlation analysis between soil properties and nitrogen and carbon cycling genes. Overall, AP and TK were strongly correlated with several nitrogen and carbon cycling genes, highlighting their role as key soil factors affecting microbial nitrogen and carbon cycling. AP was negatively correlated with genes involved in *nirA* and nitrogen uptake (*nrtA, nasF, cynA*), but positively correlated with genes related to nitrogen mineralization (*arcC*). TK showed positive correlations with nitrogen mineralization genes (*nit*, *arcC*) and dissimilatory nitrate reduction genes (*napB*), while being negatively correlated with nitrification genes (*pmoB-amoB*). In addition, TK was significantly correlated with the *PRK* gene, which is involved in aerobic carbon fixation, suggesting that potassium may be an important nutrient supporting microbial carbon fixation in cassava field soils.

### Redundancy analysis of soil physicochemical properties and the abundance of the genes involved in carbon and nitrogen cycling

Figure 8A presents the redundancy analysis (RDA) of carbon cycling genes and soil properties. Here, the two axes explained approximately 79% of the variation, and TK, AK, AP and SOM emerged as the major factors on carbon cycling genes abundance. A clear separation among groups was observed in both RDA plots. Groups treated with biochar (BB and BR) were mainly located in the positive region of RDA1, whereas Groups treated without biochar (CB and CR) were positioned on the negative side. This indicates that biochar may alter the soil microbial community structure, thereby affecting the abundance of genes involved in carbon and nitrogen cycling. Figure 8B shows the RDA of nitrogen cycling genes and soil physicochemical properties. The two axes accounted for about 74% of the total variation of nitrogen cycling genes abundance. Among the measured soil properties, TK, AK and AP exerted the stronger effects on nitrogen cycling genes abundance, which is consistent with the results of the correlation analysis.

**Figure 8:**
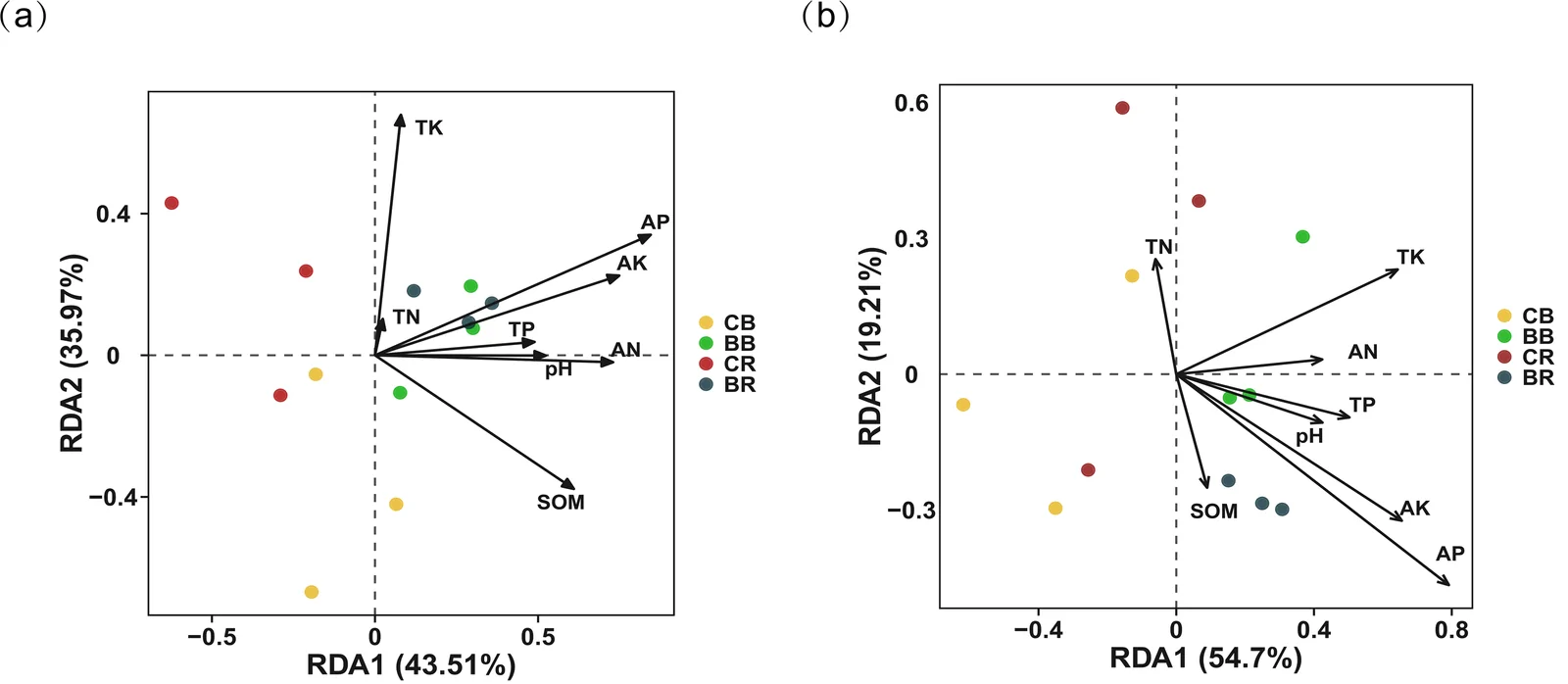
Redundancy analysis of soil physicochemical properties and carbon and nitrogen cycling genes. The RDA analysis between soil physicochemical properties and nitrogen cycling-related genes (A). The RDA analysis between soil physicochemical properties and carbon cycling-related genes (B).

## Discussion

### Biochar increased soil fertility indicators in a continuous cassava cropping system

Based on this study, biochar application was associated with improved soil physicochemical properties compared to the untreated soils (Fig. 1). Notably, soil pH and SOM increased after biochar application in cassava field soils, two commonly used indicators of soil health. Previous studies have found that continuous cassava cultivation leads to soil acidification (Zhu, Wei & Qin, 2025), which is likely linked to the heavy use of nitrogen fertilizers and the relatively low efficiency of nitrogen utilization (Zhang et al., 2022b). In this study, biochar application increased soil pH, which may have contributed to alleviating further soil acidification in the cassava field. This result was consistent with earlier research and reinforced biochar’s role in improving soil acidity (Feng et al., 2024; Zhang et al., 2018). In the Guangxi region of China, soils are generally prone to acidification. This pH increase may also promote microbial enzyme activity and nutrient cycling, creating a more supportive environment for microbial functional potential.

Biochar application also increased SOM, which is likely related to the direct input of recalcitrant carbon and the capacity of biochar surfaces to stabilize organic matter (Tang et al., 2025). Its porous structure and aromatic components not only help adsorb and stabilize organic carbon but also enhance the soil’s ability to store carbon (Afshar & Mofatteh, 2024). It should also be noted that the SOM method used in this study was able to reflect the changes in soil organic matter associated with biochar application, although this approach may not fully capture the stable carbon pool derived from biochar. As a result, the contribution of biochar-derived carbon to total soil carbon may have been underestimated. Future work should include total carbon measurements to provide a more comprehensive assessment of carbon dynamics in biochar-amended soils. Continuous cassava cultivation led to a reduction in the availability of soil nutrients, accelerating soil degradation (Zhu, Wei & Qin, 2025). In our study, biochar application significantly improved the nutrient levels in cassava field soils, with AN being highest in the non-rhizosphere soil, and AP and AK being highest in the rhizosphere. Biochar, with its unique porous structure, effectively adsorbed both cations and anions, playing a critical role in soil nutrient retention (Adhikari, Moon & Timms, 2024; Yu et al., 2024). Furthermore, previous research indicated that biochar, acting as an adsorbent, enhances nitrogen use efficiency, thereby significantly reducing nitrogen leaching (Xu et al., 2016). These improvements not only enhance nutrient retention but may also facilitate microbial-mediated carbon and nitrogen cycling, further supporting soil fertility in cassava fields. The differences in nutrient levels between the two soil types in this study may be closely tied to changes in the rhizosphere microenvironment, which is influenced by the root exudates of cassava and microbial community structure (Han et al., 2023; He et al., 2023).

### Biochar application modified the functional community structure of soil microorganisms in cassava fields under continuous cropping

In the same experimental system, previous 16S rRNA gene amplicon sequencing revealed biochar-induced changes in bacterial community composition in cassava soils (Zhu et al., 2026). Biochar increased the relative abundance of Chloroflexi and Actinobacteriota in bulk soil, whereas Cyanobacteria and Nitrospirota showed higher abundances in rhizosphere soil. These compartment-specific responses indicate that biochar may differently regulate microbial communities depending on soil microenvironments. To further explore the functional responses of soil microbial communities under different biochar application rates, functional annotation was performed using the COG, CAZyme, and KEGG databases (Cai et al., 2020). The primary KEGG annotation revealed that “metabolism” was the most enriched functional category (Fig. 3A). This highlights that microbial metabolic processes are key in both rhizosphere and bulk soils under continuous cassava cropping, which is consistent with previous findings that metabolism is a key process in microbial communities (Zhu, Wei & Qin, 2025). In this study, biochar’s effects on the microbiomes in the rhizosphere and non-rhizosphere soils were different. While biochar application had minimal effects on most functional categories in non-rhizosphere soil (Fig. 3B, Fig. S2), it significantly influenced gene abundance in certain functional categories in rhizosphere soil (Fig. 3C). Homologous recombination is a conserved mechanism that mediates genetic information exchange or repair using homologous DNA sequences, plays a central role in DNA double-strand break repair and meiosis, and contributes to the maintenance of genome stability (Choi et al., 2025). Higher abundances of genes related to “homologous recombination” and “DNA replication” categories in rhizosphere soil may reflect enhanced microbial adaptation and genome maintenance under biochar treatment.

Annotation analysis in the eggNOG database showed the top 10 functional categories with the highest gene abundance (Fig. 4A). In non-rhizosphere soil, biochar application did not significantly affect microbial functional categories (Fig. S3). However, biochar exerted stronger effects in the rhizosphere, likely owing to more dynamic plant–microbe interactions. Biochar application in rhizosphere soil significantly reduced the gene abundance in categories like “function unknown”, “cell wall/membrane/envelope biogenesis”, and “carbohydrate transport and metabolism” (Fig. 4B), while increasing the abundance of genes in the “replication, recombination, and repair” category. Replication, recombination, and repair constitute the three fundamental modes of cellular DNA processing, collectively responsible for the duplication, rearrangement, and correction of genetic material, thereby ensuring the integrity and stability of the genome (Hanawalt, 2007). Taken together, these results indicate that biochar addition was accompanied by changes in rhizosphere microbial functional gene profiles, potentially reflecting altered microbial stress responses or growth-related processes under the rhizosphere microenvironment.

Annotation analysis in the CAZyme database indicated that the “glycoside hydrolases” category was the most enriched functional category in all soil samples (Fig. 5A). Significant differences in the gene abundance between the biochar treatment and the control were observed in both soil types, with more evident changes in the rhizosphere soil. These differences in functional groups, particularly in the “glycoside hydrolases”, “glycosyl transferases”, and “carbohydrate-binding modules” categories (Figs. 5B, 5C), suggest that biochar may influence the metagenome-derived functional potential for carbohydrate degradation and related nutrient cycling. An increased abundance of glycoside hydrolase–related genes may indicate a greater capacity for the breakdown of complex carbohydrates (Kato et al., 2018; Kojima et al., 2022), which could contribute to carbon turnover and nutrient availability for the soil microbial community.

### Biochar application affected the abundance of genes related to carbon and nitrogen cycling in soils of cassava fields under continuous cropping

Biochar addition was associated with significant shifts in two carbon-cycling genes in the rhizosphere. The increased abundance of *coxA*, a key gene involved in aerobic respiration, may indicate a greater genetic potential for oxygen-dependent metabolism, which is consistent with reports that biochar can improve soil aeration and microbial habitat conditions (Case et al., 2012; Pranagal & Kraska, 2020; Zhang et al., 2023a; Zhang et al., 2022a). The lower abundance of *LDH* gene indicates that fermentation-related functions were less represented in the rhizosphere after biochar addition (Fig. 6B). This change may be related to better soil aeration and oxygen supply, as well as shifts in community composition. Combined with the increase in *coxA* gene, the gene profile is more consistent with aerobic carbon metabolism than with fermentation. This shift in functional potential may contribute to changes in carbon turnover in cassava soils.

Nitrogen cycling genes showed a stronger response to biochar in the rhizosphere than in non-rhizosphere soil. Biochar significantly increased the abundance of *norB* (denitrification) and *GDH2* (nitrogen mineralization). The gene *norB* is a key gene in the process of converting nitric oxide to nitrous oxide (Braker & Tiedjë, 2003). Soil organic matter may promote denitrification by providing carbon sources and energy for denitrifying microorganisms involved in nitrogen transformation (Chen et al., 2025). As an additional source of organic matter, biochar application may increase the relative abundance of *norB* gene. *NifK*, *nifD*, and *nifH* are key genes involved in nitrogen fixation, and biochar application significantly increased the abundance of *nifD* gene. However, no significant differences were observed in the abundance of *nifK* and *nifH* genes between the rhizosphere soils of the control group and biochar-treated group. Bamboo biochar application increased the abundance of nitrogen fixation genes during composting (Yan et al., 2022), which is consistent with the findings of this study. Our findings suggest that biochar can modify the predicted functional potential of microbial communities for nitrogen transformation and biological nitrogen fixation, which may help increase soil nitrogen availability in cassava systems. The reduction in genes abundance related to nitrogen assimilation (*gltB*), assimilatory nitrate reduction (*nirA*), nitrogen mineralization (*cynS*), and nitrogen uptake (*nrtA*, *nasF*, *cynA*, *nrtC*, and *nasD*) shows decreased microbial dependency on these pathways under biochar treatment in the cassava field (Fig. 7). This is likely attributed to the enhanced nitrogen retention and supply associated with biochar amendment (Yang et al., 2024; Zhang, Zhang & Qiu, 2022). In bulk soil, nitrogen cycling genes did not differ significantly between treatments, whereas clearer shifts were observed in the rhizosphere, possibly because root exudates and plant–microbe interactions make this microenvironment more responsive to biochar amendment.

Soil microbes play a central role in soil carbon and nitrogen cycling, and their functioning is tightly connected to the supply of key nutrients (Gao et al., 2024; Liang et al., 2025; Liu et al., 2024a). In this study, available phosphorus and total potassium stood out as the soil variables most closely associated with variation in carbon and nitrogen cycling gene profiles in cassava field soils (Fig. 8). This pattern implies that phosphorus and potassium availability may be important constraints shaping microbial processes related to carbon turnover and nitrogen transformation in this system. From a management perspective, fertilization strategies for continuous cassava cultivation should balance nitrogen inputs with adequate attention to phosphorus and potassium. However, although biochar application increased the levels of phosphorus and potassium in soil, it remains unclear to what extent these nutrients can be effectively absorbed and utilized by cassava plants, especially under long-term continuous cropping conditions.

## Conclusions

Biochar application improved soil conditions in continuous cassava cropping systems by increasing soil pH, organic matter content, and nutrient availability. Metagenomic analysis showed that biochar reshaped microbial functional potential associated with carbon and nitrogen transformation, with the rhizosphere exhibiting a more pronounced response than bulk soil. These results highlight the importance of the rhizosphere in mediating microbial responses to biochar and suggest that biochar amendment may help maintain nutrient cycling and soil functional stability under long-term cassava cultivation. Overall, this study provides new insights into the microbial mechanisms underlying biochar-mediated regulation of soil health in cassava cropping systems. Biochar may therefore represent a promising strategy for improving soil sustainability in long-term cassava production. Nevertheless, further studies integrating plant growth, nutrient utilization efficiency, and long-term field performance are still needed to better evaluate the sustainability of biochar application in cassava production systems.

## Supplemental Information

10.7717/peerj.21731/supp-1 Supplemental Information 1 Supplemental figures and tables

10.7717/peerj.21731/supp-2 Supplemental Information 2 Dataset

## Additional Information and Declarations

### Field Study Permissions

The following information was supplied relating to field study approvals (i.e., approving body and any reference numbers): This study did not involve human participants, vertebrate animals, or identifiable personal data, and therefore did not require Institutional Review Board (IRB) approval.

### Data Availability

The following information was supplied regarding data availability: The raw data are available in the Supplemental Files. The raw sequence data are available at NCBI: SRP655454.
