# Supplementary material for: Biochar application alters soil properties and microbial gene profiles in a continuous cassava cropping system
Source: PeerJ. 2026 Sep 28;14:e21731. doi: 10.7717/peerj.21731 (PMC13629565; doi:10.7717/peerj.21731)
Supplement: Supplementary file 1 [file peerj-14-21731-s001.docx]

Fig. S1 The absolute abundance of genes in different pathways related to carbon cycling in the comparison between CB and BB. CB: bulk soil without biochar application; BB: bulk soil with biochar application. NS represents no significant differences.

Fig. S2 Non-significant KEGG level 3 functional categories between CB and BB (*P* > 0.05). CB: bulk soil without biochar application; BB: bulk soil with biochar application.

Fig. S3 Non-significantly different eggNOG functional categories between CB and BB (*P* >0.05). CB: bulk soil without biochar application; BB: bulk soil with biochar application.

Fig. S4 Non-significantly different eggNOG functional categories between the CR and BR (*P* >0.05). CR: rhizosphere soil without biochar application; BR: rhizosphere soil with biochar application.

Fig. S5 The functional genes related to soil nitrogen cycling. The contents inside the oval represent

the pathways of the nitrogen cycle; genes are indicated in italicized font within rectangular boxes; arrows denote the direction of the pathways; the names of the pathways, related genes, and arrows are all shown in the same color. N_2_: nitrogen; NH_4_^+^: ammonium ion; NH_2_OH: hydroxylamine; NO_2_^−^: nitrite ion; NO_3_^−^: nitrate ion; N_2_H_4_: hydrazine; NO: nitric oxide; N_2_O: nitrous oxide.

Fig. S6 The absolute abundance of genes in different pathways related to nitrogen cycling in the comparison between CB and BB. CB: bulk soil without biochar application; BB: bulk soil with biochar application. NS represents no significant differences.

Fig. S7 Pearson correlation of soil physicochemical properties and carbon and nitrogen cycling genes. The Pearson correlation analysis between soil properties and nitrogen cycling-related genes (a). The Pearson correlation analysis between soil properties and carbon cycling-related genes (b).

** represents extremely significant differences at *P* <0.01, and *represents extremely significant differences at *P* <0.05.

Table S1. The physicochemical characteristics of the corn stalk biochar used in this study.

| raw materials | pH | ash content (%) | specific surface area(m^2^/g) | average pore diameter (nm) | organic carbon (g/kg) | total nitrogen (g/kg) | total phosphorus (mg/kg) | total potassium (g/kg) |
| --- | --- | --- | --- | --- | --- | --- | --- | --- |
| corn straw | 9.46 | 8.43 | 68 | 7.45 | 422.1 | 83.4 | 23.1 | 161.2 |

**Table S2 Overview of the soil metagenomic sequencing**

| Sample | Total reads | Clean reads | ORFs | Total length  (bp) | Average length (bp) | Max length (bp) |
| --- | --- | --- | --- | --- | --- | --- |
| CB_1 | 97,784,902 | 92,134,492 | 1,116,136 | 564,561,743 | 505.82 | 14,283 |
| CB_2 | 66,007,798 | 61,863,280 | 546,272 | 263,836,269 | 482.98 | 10,963 |
| CB_3 | 72,748,402 | 67,959,190 | 681,646 | 331,038,717 | 485.65 | 9,089 |
| BB_1 | 72,237,678 | 66,599,962 | 596,545 | 291,167,351 | 488.09 | 16,544 |
| BB_2 | 90,643,330 | 85,313,396 | 1,002,611 | 500,523,488 | 499.22 | 12,527 |
| BB_3 | 69,574,078 | 65,620,072 | 607,484 | 288,829,865 | 475.45 | 10,047 |
| CR_1 | 71,502,152 | 67,044,096 | 624,031 | 302,736,708 | 485.13 | 9,043 |
| CR_2 | 68,541,882 | 63,527,536 | 554,303 | 269,864,208 | 486.85 | 16,200 |
| CR_3 | 72,417,220 | 67,100,400 | 605,483 | 295,809,336 | 488.55 | 12,871 |
| BR_1 | 77,717,904 | 72,492,478 | 780,065 | 376148343 | 482.2 | 11,021 |
| BR_2 | 61,956,296 | 57,845,650 | 583,123 | 278,218,439 | 477.12 | 9,067 |
| BR_3 | 70,898,678 | 66,031,984 | 700,954 | 337,652,230 | 481.7 | 9,420 |
| Average | 74,335,860 | 69,461,045 | 699,887.8 | 341,698,891 | 486.56 | 11,756.25 |
